# Is there a role for blood purification therapies targeting cytokine storm syndrome in critically severe COVID-19 patients?

**DOI:** 10.1080/0886022X.2020.1764369

**Published:** 2020-05-22

**Authors:** Gang Chen, Yangzhong Zhou, Jie Ma, Peng Xia, Yan Qin, Xuemei Li

**Affiliations:** a Nephrology Department, Peking Union Medical College Hospital, Peking Union Medical College, Chinese Academy of Medical Sciences, Beijing, China; b Department of Internal Medicine, Peking Union Medical College Hospital, Peking Union Medical College, Chinese Academy of Medical Sciences, Beijing, China

**Keywords:** COVID-19, acute kidney injury, cytokine storm, blood purification, oXiris, Cytosorb

## Abstract

The coronavirus disease-19 (COVID-19) has spread over many countries and regions since the end of 2019, becoming the most severe public health event at present. Most of the critical cases developed multiple organ dysfunction, including acute kidney injury (AKI). Cytokine storm syndrome (CSS) may complicate the process of severe COVID-19 patients. This manuscript reviews the different aspects of blood purification in critically ill patients with AKI and increased inflammatory factors, and examines its potential role in severe COVID-19 treatment. Continuous renal replacement therapy (CRRT) has been practiced in many sepsis patients with AKI. Still, the timing and dosing need further robust evidence. In addition to the traditional CRRT, the high-throughput membrane with adsorption function and cytokine adsorption column are two representatives of recently emerging novel membrane technologies. Their potential in removing inflammatory factors and other toxins prospects for the treatment of severe COVID-19.

## 1. Introduction

In December 2019, a series of unexplained pneumonia cases appeared in Wuhan, a major city in China [1]. The image findings were consistent with the characteristics of viral pneumonia, and gene sequencing confirmed that it was a new type of coronavirus [1]. The new coronavirus is a single-stranded positive-strand RNA virus with 79.6% similarity to the severe acute respiratory syndrome (SARS) virus genome [2]. The International Committee on Taxonomy of Viruses officially named the virus SARS-CoV-2 in February 2020.

Much about the new disease, which is labeled coronavirus disease-19 (COVID-19) by World Health Organization (WHO), is still unsatisfactorily understood. Similar to the SARS virus, SARS-CoV-2 binds to angiotensin-converting enzyme-2 (ACE2) receptors on the cell surface through S protein [2]. The basic reproductive number (R_0_) of COVID-19 ranged from 2 to 3.5 at the early phase regardless of different prediction models, which was higher than SARS and Middle East Respiratory Syndrome (MERS) [3]. Until the end of April 2020, the virus has spread to every continent except Antarctica, with cumulatively more than 2 million confirmed COVID-19 cases, becoming the currently most severe public health event [4,5].

## 2. Severe COVID-19 and kidney impairments

The typical clinical manifestations of COVID-19 include fever and respiratory symptoms, but 13.8–25.5% patients will develop into severe cases [6,7], and about 5–6% will need intensive care unit (ICU) admission or mechanical ventilation [8,9]. Due to its extremely contagious nature, many severe patients have appeared in the short term. At present, some substantial case analysis indicated that the overall mortality of COVID-19 was about 1.36–3.46% [1,7,8].

According to the national guidelines published by the Chinese National Health Commission [10], severe cases are featured by either: (1) respiratory rate >30/min, or (2) oxygen saturation ≤93%, or (3) PaO_2_/FiO_2_ ratio ≤300 mmHg. The critically severe cases are diagnosed by either: (1) respiratory failure requiring mechanical ventilation, or (2) shock, or (3) combined other organ failure and needed ICU admission. Not merely suffered from diffuse alveolar damage and acute respiratory failure, severe patients also endure multiple-organ involvements such as gastrointestine [11], coagulation [12], and kidney [13]. In a sizeable COVID-19 cohort, acute kidney injury (AKI) occurred in 5.1% of patients [13], while AKI involvement increased to as high as 29% in a large cohort of 52 critically severe COVID-19 patients [9]. COVID-19 companied with AKI usually resulted in significantly higher mortality [14]; one study demonstrated AKI stage 3 was associated with a 4.72 times hazard risk for in-hospital death [13].

Except for AKI, proteinuria occurs in 43.9–63% of COVID-19 patients [13,15]. The kidney imaging has shown a significantly lower CT value compared with patients without nephropathy [15], indicating the renal parenchymal inflammation and edema.

## 3. Severe COVID-19 and cytokine storm syndrome

Cytokine storm syndrome (CSS), featured by an overactive immune response and unsustainable cytokine release [16], is now increasingly suspected in the pathogenesis of severe COVID-19 [17–19]. A sizable part of patients with COVID-19 has mild clinical symptoms at the early stage of infection. Still, the disease may rapidly deteriorate in the later stage manifested with acute respiratory distress syndrome (ARDS) and followed by multiple organ dysfunction syndromes (MODS) [1,8,20]. These signs suggest that immune disorders and cytokine storms may play an essential role in the organ damage of severe COVID-19 [21].

During COVID-19 infection, 83.2% of patients presented lymphopenia, and the decrease degree was related to disease severity [8]. Further functional studies have confirmed that the counts of CD4 and CD8 positive T cells significantly reduced, but in an overactive state [22]. Compared with the mild patients, the serum levels of inflammatory factors in severe patients were considerably increased, including interleukin (IL)-6, tumor necrosis factor α (TNF-α), IL-2, monocytes Chemokine-1 (MCP-1), macrophage inflammatory protein 1α (MIP1A), etc. [1]. Pathology of the lung tissue in the deceased patients showed diffuse alveolar injury with cell fibrous mucus-like exudate, infiltrating with lymphocyte dominated inflammatory cells [22]. This manifestation resembled the pathological characteristics of ARDS in SARS and MERS [22]. Another pathological lung finding revealed that alveolar macrophages with SARS-CoV-2 infection were expressing ACE2, suggesting the role of macrophages as direct host cells of SARS-CoV-2 and potential drivers of CSS in COVID-19 [17].

Cytokine storm syndrome refers to the excessive release of cytokines in response to external stimuli and is an essential cause of ARDS and MODS [23,24]. In addition to severe infections, autoimmune diseases, graft-versus-host disease, tumors, and tumor immunotherapy (CART therapy and immune checkpoint inhibitors) also trigger CSS. The severe infections in SARS [25], H5N1 [26], and H7N9 [27] have shown evidence of CSS. The CSS develops in two steps: primary response and secondary response. The primary response refers to the activation of the innate immune system following the virus invasion in alveolar epithelial cells, which initiates a rapid antiviral signaling cascade. The infected epithelial cells, innate immune cells, and endothelial cells produce a variety of cytokines to prevent the spread and replication of the virus. Meanwhile, the effector cells are also recruited to accelerate the apoptosis of infected cells and promote tissue repair. Further cascade generating more cytokines is called secondary cytokine storms [28]. Usually, the anti-inflammatory response and tissue repair processes are mild and self-limiting. However, if the cytokines, such as TNF-α, IL-6, IL-1, and IFN, continue to increase, they can stimulate pathological reactions such as diffuse alveolar damage, transparent membrane formation, and pulmonary fibrosis [29]. Cytokines, when entering the circulation, can induce extensive endothelial dysfunction, disseminated intravascular coagulation, and MODS [23].

There is currently no standard treatment for CSS. In severe cases of SARS, H1N1, and H7N9 virus infections [30,31], glucocorticoids have been administrated. However, the efficacy has not been confirmed, and there is a concern of increased mortality in patients who are more likely to develop ventilator-associated pneumonia [32]. In the CSS secondary to CART therapy, IL-6 receptor monoclonal antibodies can effectively inhibit inflammation, which supports the critical pathogenic role of IL-6 in some specific CSS [33]. However, there has been no systematic study on CSS secondary to severe SARS-CoV-2 infection.

## 4. Potential role of blood purification therapies for CSS in severe COVID-19

### 4.1. Continuous renal replacement therapy

The national guidelines published by the Chinese National Health Commission [10] proposed blood purification therapies for COVID-19 patients with a high inflammatory response. Based on the evidence of increased cytokines and imagings that suggest inflammation, some studies have recommended early intervention with continuous renal replacement therapy (CRRT) and immunoadsorption [15]. The application of CRRT in patients with severe MERS has proven effective [34]. In addition to improving the overload water status, it also benefits patients in the aspect of removing inflammatory factors [35].

CRRT may help improve the prognosis of critically ill patients, but there are also conflicting opinions. In terms of timing, the ELAIN randomized controlled trial (RCT) suggested that implement of CRRT in patients within 8 h following AKI stage 2 showed a significantly lower mortality rate within 90 days, compared with patients receiving CRRT within 12 h after AKI stage 3 diagnosis [36]. However, another multicenter RCT did not find the improvement in 90-day mortality in such patients with early CRRT [37]. In terms of CRRT dosage, an early study claimed that high-volume hemofiltration with an ultrafiltration rate of 45 mL/kg/h could improve the prognosis in the severe AKI patients [38]. However, it should be noted that in several prospective studies involving patients with sepsis, the analysis did not reveal advantages for high-volume hemofiltration (ultrafiltration rate 35–40 mL/kg/h) [39,40]. The IVOIRE study also indicated that CRRT with an ultrafiltration rate of 70 mL/kg/h did not improve the 28-day mortality rate in septic patients [41].

There may be some explanations for the contradictory evidence of CRRT in severe septic patients. First, the heterogeneity of such patients is substantial, which dilutes the validity of the conclusions of the study. Therefore, the determination in optimal timing and dosing of CRRT in severe sepsis patients still calls for large multicenter RCTs with better design [42]. Second, we may need to consider early CRRT intervention and ensure adequate treatment doses simultaneously. Third, many inflammatory factors, including TNF-α, IL-6, and IL-1, are mostly macromolecules. Before attempting to remove these factors, an evaluation of the filtration coefficient of a specific filtration membrane is required. High cutoff (HCO) membrane can effectively remove macromolecules of 20–60 kD [43,44]. Early research suggested that it could effectively improve peripheral blood lymphocyte count and reduce circulating IL-6 levels, but the clearance of TNF-α was limited [43,44]. However, in a recent phase II double-blinded study, HCO hemofiltration treatment in patients with septic shock did not significantly improve mortality [45].

Although there are diverse conclusions on the application of CRRT in patients with sepsis, previous research has shed light on blood purification therapies in severe COVID-19. First, previous studies have confirmed the safety of blood purification in critical patients with unstable circulatory status, suggesting that similar treatments are applicable for severe COVID-19 [39–41]. Besides, they remind us to systematically monitor the inflammatory status of severe COVID-19 patients, determine the clearance level of crucial inflammatory factors, and consider the combination of other dialysis modes in addition to conventional CRRT.

### 4.2. Potential value of high-throughput membrane with adsorption function

In recent years, some high-throughput membranes with high adsorption levels have emerged, which can improve the removal of medium and high molecular weight solutes through ionic charge interactions. For example, the AN69 membranes have a highly hydrophilic hydrogel structure, and *in vitro* experiments have confirmed that they can effectively adsorb inflammatory mediators [46,47]. Among them, oXiris is a novel iterative product based on the AN69 membrane. By modifying the surface with a multilayer linear structure of polyethyleneimine cationic polymer, oXiris can additionally adsorb endotoxins with negative charges on the surface [47]. A prospective case-control study in Hong Kong found that after 48 h of oXiris treatment, the scores of sequential organ failure assessment (SOFA) had improved significantly, compared to the historical control group (37% reduction vs. 3% increase) [48]. A multicenter retrospective study in France enrolled 31 patients with sepsis and AKI. After the oXiris treatment, the in-hospital mortality was significantly reduced by about 30%, and the acidosis status and lactic acid levels were also considerably improved in patients [49]. Other summaries also indicated similar evidence, and the earlier application of the oXiris membrane seems to be more prominent [50,51]. The oXiris membranes have been marketed in Europe, and previous reports have not concluded severe complications. With the special heparin-coated design in the column, oXiris can be used without anticoagulation for patients with an increased risk of bleeding [51].

### 4.3. Potential value of cytokine adsorption column

The coupled-plasma filtration adsorption (CPFA) technology can separate plasma through a specific column during extracorporeal circulation to realize cytokine adsorption. The COMPACT study planned to include 330 patients with septic shock and verify the efficacy of 5-day CFPA treatment, but the trial was terminated due to no improvement in mortality after the enrollment of 192 patients [52]. Few studies have so far focused on the evaluation of prognostic improvement in patients with sepsis by adsorption of inflammatory factors. Cytosorb is a novel device utilizing biocompatible porous polymer adsorbent microbeads. It was first marketed in Europe in 2011 and was approved for the removal of inflammatory factors, bilirubin, and myoglobin. Cytosorb can be simultaneously applied with the standard hemodialysis, hemofiltration, and extracorporeal membrane oxygenation (ECMO). Several retrospective studies have confirmed that Cytosorb can effectively reduce the levels of inflammatory factors and significantly improve the mortality in severe patients with septic shock [53,54]. To date, it has been used for over 73,000 times in more than 800 clinical settings worldwide, with excellent tolerance and safety profiles. In patients with severe COVID-19, the application of effective cytokine adsorption may improve the prognosis of patients at the early stage of the CSS.

### 4.4. Timing for blood purification therapies in severe COVID-19

There may be a ‘windows of opportunity’ for severe COVID-19 patients. The combination of higher levels of IL6 (>24.3 pg/mL) and D-dimer (>0.28 μg/L) were predictive of the development of severe pneumonia in COVID-19 patients, with a sensitivity of 93.3% and a specificity of 96.4% [1,55]. The median time from disease onset to ICU admission was10.5 days, after a median of 1.5 days from ARDS diagnosis and 2.5 days from dyspnea onset [1]. The half-life of inflammatory factors in circulation is only a few minutes, suggesting that blood purification treatment targeting inflammatory factors should be considered in the cases at an early stage. After the cascade effect of inflammatory factors, the removal efficiency may be limited [56,57]. Early application of blood purification therapies with intensive dose in severe COVID-19 patients may achieve better efficacy and realize therapeutic goals such as stabilizing hemodynamics and improving MODS.

## 5. Prospects

COVID-19 is a new disease with severe cases that may complicate with CSS. Based on the real-world experience derived from other virus-medicated sepsis, such as SARS and MERS, blood purification therapies have a potential role in the treatment of severe COVID-19 combined with AKI. Some new membrane technologies targeting cytokine removal may result in better outcomes. There may be a ‘window of opportunity’ for patients with severe COVID-19, and early application of blood purification therapies should be considered.
